# ERRα regulates the growth of triple-negative breast cancer cells via S6K1-dependent mechanism

**DOI:** 10.1038/sigtrans.2017.35

**Published:** 2017-08-25

**Authors:** Adi Y Berman, Subrata Manna, Naomi S Schwartz, Yardena E Katz, Yang Sun, Catherine A Behrmann, Jane J Yu, David R Plas, Anya Alayev, Marina K Holz

**Affiliations:** 1 Department of Biology, Yeshiva University, New York, NY, USA; 2 Division of Pulmonary, Critical Care, and Sleep Medicine, Department of Internal Medicine, University of Cincinnati College of Medicine, Cincinnati, OH, USA; 3 Department of Cancer Biology; University of Cincinnati, Cincinnati, OH, USA; 4 Department of Molecular Pharmacology and the Albert Einstein Cancer Center, Albert Einstein College of Medicine, Bronx, NY, USA

## Abstract

Estrogen-related receptor alpha (ERRα) is an orphan nuclear factor that is a master regulator of cellular energy metabolism. ERRα is overexpressed in a variety of tumors, including ovarian, prostate, colorectal, cervical and breast, and is associated with a more aggressive tumor and a worse outcome. In breast cancer, specifically, high ERRα expression is associated with an increased rate of recurrence and a poor prognosis. Because of the common functions of ERRα and the mTORC1/S6K1 signaling pathway in regulation of cellular metabolism and breast cancer pathogenesis, we focused on investigating the biochemical relationship between ERRα and S6K1. We found that ERRα negatively regulates S6K1 expression by directly binding to its promoter. Downregulation of ERRα expression sensitized ERα-negative breast cancer cells to mTORC1/S6K1 inhibitors. Therefore, our results show that combinatorial inhibition of ERRα and mTORC1/S6K1 may have clinical utility in treatment of triple-negative breast cancer, and warrants further investigation.

## Introduction

Estrogen-related receptor alpha (ERRα) is an orphan nuclear receptor and an important component of signaling networks in breast cancer cells.^1^ ERRα is considered to be a master regulator of cellular energy metabolism as it regulates transcription of various enzymes involved in glycolysis, tricarboxylic acid cycle, lipid, amino- and nucleic-acid metabolism. ERRα shares a high degree of identity with estrogen receptor alpha (ERα), however, their expression, mode of activation and biological functions are different across various tissue types.^2^ ERRα is a critical regulator of cancer development because it can accommodate energy demands of proliferating cancer cells.^3–6^

Consistent with its role in cellular metabolism, mice deficient in ERRα have increased metabolism, are resistant to diet-induced obesity and are unable to adapt to cold temperatures.^7–9^ In particular, ERRα is necessary for response to physiological stress as well as for fate and determination of myocytes, adipocytes, T cells, osteoblast and intestinal epithelia.^10–14^

Since ERRα shares a high degree of homology with ERα, it can regulate its cognate genes through binding to estrogen response elements that are usually bound by ERα. However, *in* vivo studies have shown that ERRα also binds to a unique motif termed the estrogen-related response element,^4,15^ indicating that ERα and ERRα can co-regulate a subset of common target genes in addition to regulation of their unique target genes.

Clinically, ERRα is highly expressed in ovarian, cervical, colorectal and prostate tumors and is associated with more aggressive tumors. In breast and ovarian cancer patients, ERRα expression tends to be inversely correlated with the expression of ERα and progesterone receptor, and high ERRα expression was shown to associate with poor prognosis and increased rate of recurrence.^3,16–18^ Moreover, studies have shown that downregulation of ERRα activity by pharmacological antagonists decreased cell proliferation and tumorigenicity in both ERα-positive and ERα-negative breast cancers.^19–21^ In addition, knockdown of ERRα expression using short hairpin RNA significantly reduced the growth rate of xenograft tumors,^3^ and it was shown that genetic deletion of *Esrra* significantly delays tumor development in a mouse model of ERBB2-initiated mammary tumorigenesis,^22^ indicating that pharmacological regulation of ERRα may be beneficial for patients.

There exists a genetic and biochemical crosstalk between ERRα and the mechanistic target of rapamycin complex 1 (mTORC1). mTORC1 is a critical regulator and integrator of multiple cellular signals, such as growth factors, hormones, mitogens, nutrients and other signals, in regulation of cellular anabolic processes.^23^ mTORC1 is acutely sensitive to inhibition by rapamycin, an immunosuppressive and anti-neoplastic agent. Rapamycin analogs, called rapalogs, are FDA-approved for several indications, including breast cancer.^24^ PGC1α, one of the major coactivators of ERRα, affects tumor cell metabolism in skeletal muscles and kidney angiomyolipomas through the phosphatidylinositol-3 kinase (PI3K)-mTOR dependent activation of the transcription factor YY-1.^25,26^ In addition, mTOR binds to regulatory regions of genes controlled by ERRα that are involved in the tricarboxylic acid cycle and lipid biosynthesis.^27^ mTOR also regulates ERRα degradation via transcriptional control of the ubiquitin–proteasome pathway,^27^ further linking mTORC1 signaling pathway to ERRα.

The 40S ribosomal S6 kinase 1 (S6K1) is the best-characterized kinase downstream of mTORC1.^23,28^ S6K1 regulates cell size and cell cycle progression by phosphorylating several proteins controlling nucleotide biosynthesis, RNA processing, protein transcription and translation.^28^ S6K1 is encoded by the *RPS6KB1* gene localized to the chromosomal region 17q23. The 17q23 region is amplified in several breast cancer cell lines and in 10–30% of primary tumors, resulting in S6K1 overexpression.^28,29^ We have previously shown that estrogen-activated ERα positively regulates S6K1 expression by a GATA-3-dependent mechanism in mammary epithelia.^30^ ERα regulation of S6K1 expression creates a positive feedforward loop, leading to activation of ERα by S6K1, potentiating ERα-positive breast cancer cell growth.

In our previous work, we identified high ERRα expression as a biomarker of response to tamoxifen in triple-negative breast cancers (TNBC),^18^ a finding that may provide clinical benefit to this population of patients. Because ERRα can bind the same promoters as ERα, and because we found an association between high ERRα expression and a worse prognosis in breast cancer patients in our recent study,^18^ we sought to investigate whether ERRα affects the expression of S6K1 in breast cancer cells. Moreover, we investigated the correlation between ERRα expression and sensitivity to mTOR and S6K1 inhibition in breast cancer cell lines and mouse models. We found that ERRα directly binds to the S6K1 promoter and inhibits its expression, thus modulating sensitivity to mTORC1 and S6K1 inhibition.

## Materials and methods

### Cell culture

MCF7, MDA-MB-231 and MDA-MB-468 cells were grown in Dulbecco’s modified Eagle’s medium (DMEM) with 10% fetal bovine serum (FBS) and 1% penicillin–streptomycin. MDA-MB-436 cells were grown in Ham’s media with 10% FBS and 1% penicillin–streptomycin. All cells were cultured in 37 °C incubator with humidified 5% CO_2_ atmosphere. Stable ERRα knockdown and control MDA-MB-231 luciferase-expressing cells were a generous gift from Donald P McDonnell (Duke University, Durham, NC, USA). As indicated, cells were treated with either 20 nM rapamycin, 20 μM PF4708671 or 5 μM XCT 790.

### Immunoblotting

Cells were lysed, and cell extracts were resolved using sodium dodecyl sulfate polyacrylamide gel electrophoresis and transferred onto nitrocellulose membrane for immunoblotting and near-infrared detection using IRDye-conjugated secondary antibodies (LI-COR, Lincoln, NE, USA) as previously described.^31^ Antibodies against S6K1, p-S6K1 T389, ERRα, p-S6 S240/244, p-S6 S235/236, ERK, 4EBP1, survivin, mcl1 and p-eIF4B S422 were purchased from Cell Signaling Technologies (Danvers, MA, USA). Actin and ERα antibodies were purchased from Santa Cruz Biotechnology (Dallas, TX, USA). GAPDH antibody was purchased from Origene (Rockville, MD, USA).

### siRNA transfection

For siRNA transfection, cells were plated at 80% confluence and transfected using Lipofectamine RNAiMAX Transfection Reagent (Invitrogen, Carlsbad, CA, USA) according to the manufacturer’s protocol. The following day, fresh media was added and cells were treated with rapamycin as indicated. si-control (NC-1) and si-ERRα (HSC.RNAi.N004451.12.1) were ordered from Integrated DNA Technologies (Coralville, IA, USA).

### Real-time RT-PCR

Total RNA was isolated using the Qiagen RNAeasy kit. For real-time reverse transcription PCR, cDNA was prepared and amplified using the iScript kit and IQ-SYBR Green in CFX96 thermal cycler (Bio-RAD, Hercules, CA, USA) following the manufacturer’s instructions. The primers used for cDNA amplification were S6K1 forward: 5′- CTCTGAGGATGAGCTGGAGG-3′, reverse: 5′- TTCTCACAATGTTCCATGCC-3′; actin forward: 5′- AATGTGGCCGAGGACTTTGATTGC-3′; reverse 5′- AGGATGGCAAGGGACTTCCTGTAA-3′. Expression of S6K1 relative to actin was analyzed as previously described.^30^

### Reporter gene assays

Luciferase reporters for *renilla*-pS2 promoter control and the *firefly-RPS6KB1* promoter were previously described.^30^ FLAG-ERRα plasmid was obtained from Donald P McDonnell (Duke University) via Addgene. Cells were transfected using Fugene HD transfection reagent (Promega, Madison, WI, USA) according to the manufacturer’s protocol with the expression plasmid or empty vector control. At 48 h post transfection, relative luciferase activity in cell lysates was detected using the dual luciferase reporter assay system and Glomax 20/20 luminometer (Promega).

### Chromatin immunoprecipitation (ChIP)

MCF7 cells were transfected with FLAG-ERRα, grown in phenol red-free DMEM with charcoal-stripped 10% FBS for 48 h, and serum-starved in phenol red-free media for 24 h in order to avoid the interference of estrogen and endogenous ERRα. ChIP was performed using ChIP kit from Millipore (Billerica, MA, USA) with antibodies for FLAG (Sigma, St Louis, MO, USA) and histone H3 (Abcam, Cambridge, UK) and ‘no antibody controls’. Relative DNA enrichment was determined as previously described.^30^

### Cell proliferation assays

Cells were seeded at a density of 2500 cells per well in 96-well plates and allowed to attach overnight. The following day, cells were placed in media supplemented with either 1% FBS or in low glucose media and treated with either 20 nM rapamycin, 20 μM PF4708671, 5 μM XCT 790, alone or in combination. Neutral red assay to measure living cells was performed 2 or 5 days post treatment as previously described.^32,33^

### Wound healing assay

Cells were seeded in 12-well plates in complete DMEM media and grown to confluency in monolayer overnight. The following day, cells were pretreated with either 20 nM rapamycin or 20 μM PF4708671 for 8–10 h and wound/scratch was created along the diameter of each well using a 200 μl pipette tip. Cell debris were removed, followed by addition of fresh media containing 20 nM rapamycin or 20 μM PF4708671. Wound healing was measured 14 h later. The experiment was performed in triplicates, and for each imaged area three different measurements were taken. Cells were imaged using EVOS FL Auto microscope under ×10 magnification. Statistical analysis was performed using two-tailed Student’s *t*-test.

### Focus formation assay

Cells were seeded at a concentration of 250 cells per well in a six-well plate in DMEM supplemented with 10% FBS and allowed to attach overnight. The next day, the media was replaced with DMEM containing 1% FBS and indicated cells were treated with 20 nM rapamycin. Fresh media was added every 3–4 days. After 10 days of treatment, cells were fixed in methanol for 10 min at −20 °C and stained using 0.5% crystal violet solution/25% methanol for 10 min at room temperature. Excess dye was washed off with water. Colonies containing 50 or more cells were counted. Statistical analysis was performed using two-tailed Student’s *t*-test.

### Animal studies

All animal work was performed in accordance with protocols approved by the Institutional Animal Care and Use Committee-Children’s Hospital Boston. Female CB17 SCID mice were purchased from Taconic. MDA-MB-231 cells-LUC control cells or MDA-MB-231 cells-LUC cells with stable ERRα knockdown were collected, suspended in 100 μl PBS (5×10^6^ ml^−1^), and injected intravenously into mice (*n*=5 per group). Lung colonization was monitored using bioluminescent live imaging at 1, 6 and 24 h post-cell injection. Ten minutes prior to imaging, animals were injected with D-luciferin (Perkin Elmer, 120 mg kg^−1^, i.p.). Bioluminescent signals were recorded using the Xenogen IVIS 200 System. Total photon flux of chest area was analyzed. Mice were administrated with vehicle or rapamycin (6 mg kg^−1^, i.p.) after imaging at 1-h post-cell inoculation. The photon flux at the chest regions was evaluated.

### Statistical analysis

All cell culture data are presented as mean±s.d. and *n*=3. Statistical significance was determined by paired Student’s *t*-test using Microsoft Excel.

## Results

**Figure 1:**
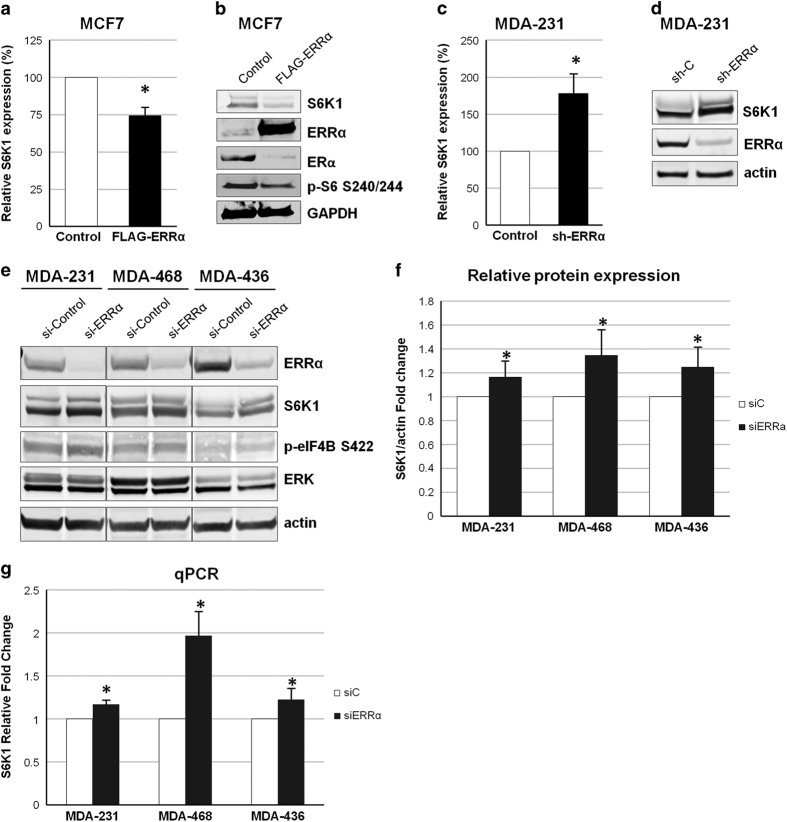
ERRα regulates S6K1 expression in ERα-positive and ERα-negative cells. (**a**) MCF7 cells were transfected with FLAG-ERRα. Forty-eight hours following transfection, cells were lysed and mRNA was prepared and analyzed for S6K1 expression using RT-qPCR as described in ‘Materials and Methods’ section. Statistical analysis was performed using paired Student’s *t-*test. * represents *P*<0.05. (**b**) Cells were transfected as in **a** and analyzed for expression of S6K1, ERRα, ERα and p-S6 S240/244 by immunoblotting. Anti-GAPDH antibody was used as loading control. (**c**) Stable MDA-MB-231 control or ERRα knockdown cells were analyzed for S6K1 expression using RT-qPCR. Statistical analysis was performed using paired Student’s *t-*test. * represents *P* <0.05. (**d**) Stable MDA-MB-231 control or ERRα knockdown cells were lysed and analyzed using the indicated antibodies. (**e**) MDA-MB-231, -468 or -436 cells were transfected with siRNA against ERRα or scrambled control. Forty-eight hours following transfection, cells were lysed and probed with the indicated antibodies. (**f**) Cells were treated as in **e** and relative S6K1 protein levels were measured using Odyssey Image Studio Ver5.2 software. Statistical analysis was performed using paired Student’s *t-*test. * represents *P*⩽0.05. (**g**) MDA-MB-231, -468 or -436 cells were treated as in **e** and analyzed for S6K1 mRNA expression using RT-qPCR. Statistical analysis was performed using paired Student’s *t-*test. * represents *P*<0.05.

To investigate the relationship between ERRα and mTORC1/S6K1 signaling pathway, we focused on ERRα regulation of S6K1 expression in ERα-positive as well as ERα-negative breast cancer cells. In ERα-positive MCF7 cells that have high ERα expression and low ERRα expression, overexpression of ERRα resulted in downregulation of S6K1 mRNA and protein levels (Figures 1a and b), as well as reduction of phosphorylation of S6 on S240/244, indicating downregulation of S6K1 activity as a consequence of reduced expression. Overexpression of ERRα also caused marked downregulation of ERα consistent with previously published results (Figure 1b).^5^ We next investigated S6K1 expression in ERα-negative, MDA-MB-231 breast cancer cells with stable knockdown of ERRα. Compared to control, MDA-MB-231 cells with reduced ERRα expression exhibited marked upregulation of S6K1 mRNA expression (Figure 1c), and S6K1 protein (Figure 1d).

We next analyzed S6K1 expression upon reduction of ERRα expression in a panel of ERα-negative breast cancer cells. Knockdown of ERRα in MDA-MB-231, -468 and -436 cells resulted in a corresponding increase of S6K1 protein levels as well as upregulation of phosphorylation of another S6K1 target protein eIF4B at S422, consistent with increased S6K1 activation (Figures 1e and f). This effect is specific to S6K1 as levels of ERK, an effector of the MAPK pathway remained unchanged upon ERRα knockdown (Figure 1e). Increases in both the p70 and p85 isoforms of S6K1 proteins were comparable in all samples, suggesting that the effects of ERRα were mediated through regulation of mRNA levels. Consistent with this, we observed that S6K1 mRNA levels were significantly upregulated upon ERRα knockdown in three ERα-negative cell lines (Figure 1g).

**Figure 2:**
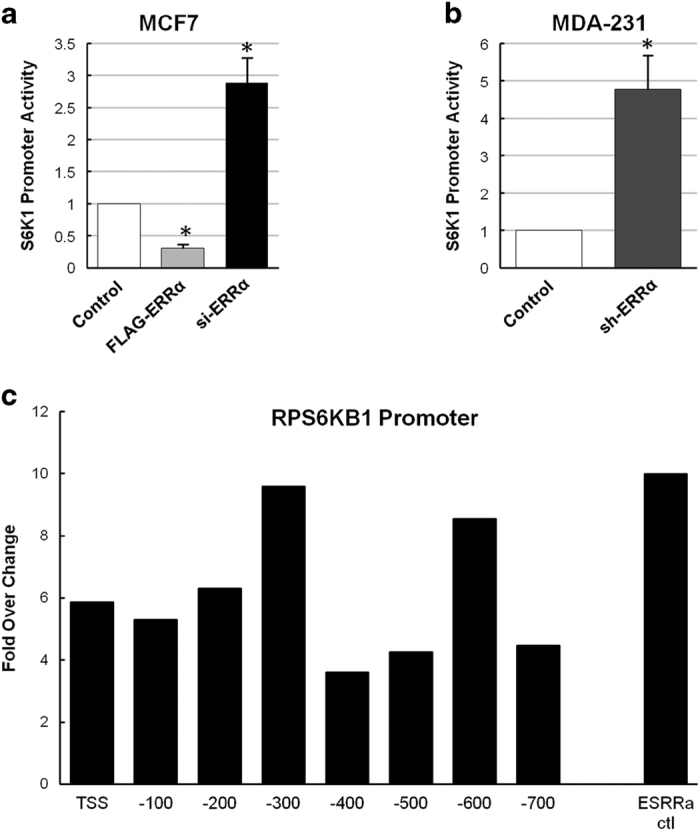
ERRα directly regulates S6K1 expression. (**a**) MCF7 cells were co-transfected with either FLAG-ERRα or siRNA targeting ERRα, the firefly luciferase S6K1 promoter reporter and *Renilla* luciferase control. Forty-eight hours post transfection, cells were lysed, and firefly luciferase expression was measured and normalized to control *Renilla* luciferase. (**b**) Stable MDA-MB-231 control cells or ERRα knockdown cells were co-transfected with FLAG-ERRα or siRNA targeting ERRα, the firefly luciferase S6K1 promoter reporter and *Renilla* luciferase control and analyzed as in **a**. (**c**) ChIP analysis of *RPS6KB1* promoter occupancy in MCF7 cells was performed as described in ‘Materials and methods’ section. ERRα was immunoprecipitated using FLAG antibody and DNA enrichment was performed using RT-qPCR with primers spaced 100 bp relative to the transcription start site. Fold-over change was calculated with respect to no antibody control and normalized to histone H3 in each sample. ERRα promoter (*ESRRA*) was used as a positive control. All statistical analysis was performed using paired Student’s *t-*test. * represents *P* <0.05.

To confirm that ERRα transcriptionally regulates S6K1 expression, we investigated whether ERRα expression affects the promoter activity of S6K1. We have previously found that the promoter region of S6K1 contains several putative estrogen response elements that may mediate ERRα interaction.^30^ Using luciferase reporter assay, we observed that in MCF7 cells that have low endogenous ERRα levels, overexpression of ERRα inhibited S6K1 promoter activity; conversely, knockdown of ERRα increased S6K1 promoter activity (Figure 2a). Similarly, in MDA-MB-231 cells with a stable knockdown of ERRα, we observed an increase in S6K1 promoter activity (Figure 2b). These results are in agreement with the S6K1 expression data described in Figures 1a and c. In addition, we wanted to demonstrate direct binding of ERRα to the S6K1 promoter using ChIP assay. Analysis of the ERRα binding to the S6K1 promoter in MCF7 cells revealed two ERRα-binding peaks ~300 and 600 bp away from the transcription start site (Figure 2c), corresponding to the approximate location of the imperfect palindrome and an estrogen response element half-site within the *RPS6KB1* promoter, as previously described.^30^ Thus, our results indicate that ERRα directly binds to the S6K1 promoter.

**Figure 3:**
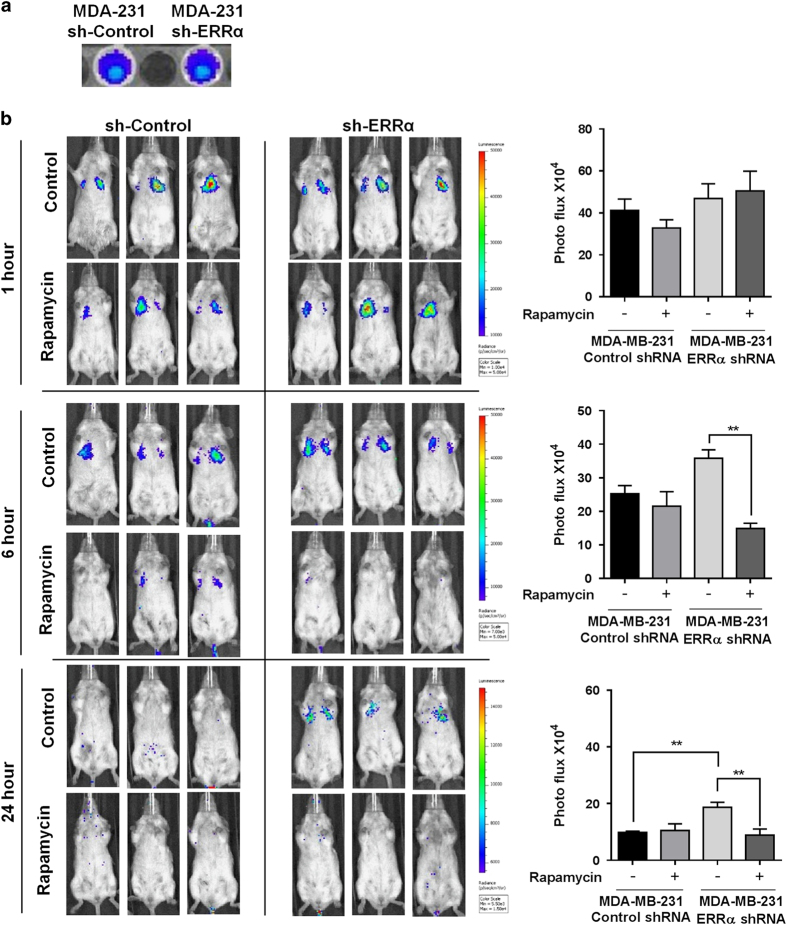
Rapamycin prevents metastasis of MDA-MB-231-sh-ERRα cells *in vivo.* (**a**) Representative bioluminescent images of MDA-MB-231 sh-Control and sh-ERRα cells. The total flux (photons per second) of cells is illustrated. (**b**) MDA-MB-231-luciferase-expressing cells were inoculated intravenously. Representative bioluminescent images of lung colonization at 1, 6 and 24 h post-cell injection are shown. The levels of bioluminescent intensity (total photon flux per second) present in the chest regions were quantified and compared between control^18^ and treatment groups. Mice were administrated with vehicle or rapamycin (6 mg kg^−1^, i.p.) 1 h post-cell inoculation. * *P*<0.05, ** <0.01, Student’s *t*-test.

To determine the impact of ERRα on the sensitivity to rapamycin in a lung colonization model of breast cancer cells, immunodeficient mice were inoculated with luciferase-expressing MDA-MB-231 with stable knockdown of ERRα or MDA-MB-231 control short hairpin RNA cells. The level of bioluminescence was measured using the Xenogen IVIS System (Figure 3a). Within 1 h post-cell inoculation, similar levels of bioluminescence were observed in the chest regions of all mice (Figure 3b). At 6 h, lung colonization of MDA-MB-231 ERRα knockdown cells was significantly reduced by rapamycin and this effect was maintained at 24 h, whereas the levels of bioluminescence in the chest regions were 2.5-fold higher in mice inoculated with MDA-MB-231 sh-ERRα cells compared with ones injected with control cells (Figure 3b and Manna *et al.*^18^).

**Figure 4:**
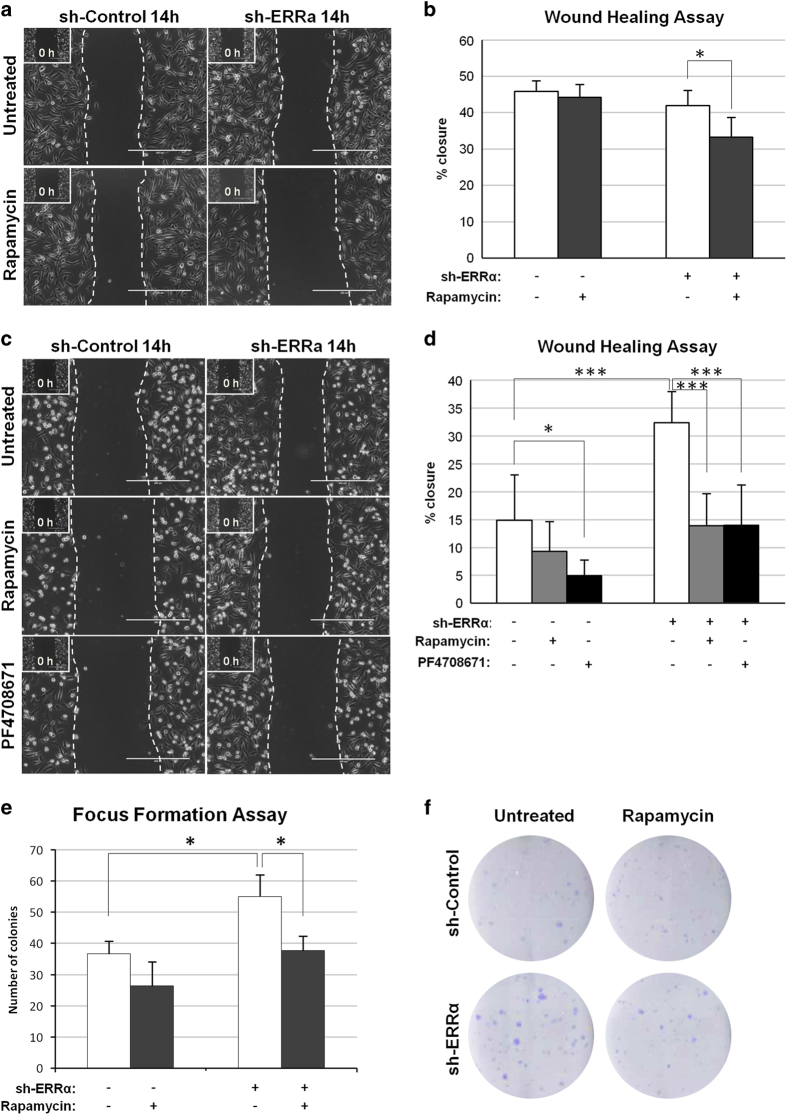
Modulation of S6K1 activity affects cell migration and transforming ability of cells with stable ERRα knockdown. (**a**) Stable MDA-MB-231 control or ERRα knockdown cells were seeded in 12-well plate in complete DMEM media and grown to confluency in monolayer overnight. The following day, cells were placed in reduced (1%) serum media and following 8–10 h of pretreatment with 20 nM rapamycin, wound/scratch was generated. Wound closure was measured 14 h post incubation (**b**) Quantification of the wound healing assay from **a** was performed and graphed using paired Student’s *t-*test. * represents *P*<0.05. ** represents *P*<0.01. *** represents *P*<0.001. (**c**) Stable MDA-MB-231 control or ERRα knockdown cells were seeded in 12-well plate in complete DMEM media and grown to confluency in monolayer overnight. The following day, cells were placed in low glucose media and following 8–10 h of pretreatment with either 20 nM rapamycin or 20 μM PF4708671, wound/scratch was generated. Wound closure was measured 14 h post incubation. (**d**) Quantification of the wound healing assay from **c** was performed and graphed using paired Student’s *t-*test. * represents *P*<0.05. ** represents *P*<0.01. *** represents *P* <0.001. (**e**) Stable MDA-MB-231 control cells or ERRα knockdown cells were seeded in six-well plate in complete DMEM media, and allowed to attach overnight. The media was then changed to reduced serum DMEM, and cells were treated with 20 nM rapamycin for 10 days. Foci were subsequently fixed with methanol and stained with crystal violet. Colonies with >50 cells were counted and graphed using paired Student’s *t-*test. * represents *P*<0.05. (**f**) Cells were treated as described in **e** and images were acquired using EVOS FL Auto microscope under ×4 magnification.

We next set out to investigate whether regulation of S6K1 expression by ERRα results in increased S6K1 activity and, subsequently, increased sensitivity to S6K1 inhibition. We have previously shown that increased S6K1 expression and/or activity, specifically under conditions that render cells exquisitely dependent on the activity of S6K1 for proliferation and survival, such as reduced serum or low glucose conditions, causes increased sensitivity to S6K1 inhibition.^32,34^ Therefore, we tested the migratory capacity of ERRα knockdown cells, treated with rapamycin or the S6K1 inhibitor PF4707671, in a wound healing assay under conditions of S6K1 pathway activation. MDA-MB-231 control or ERRα knockdown cells were grown in reduced serum media and wound healing assay was performed following 14 h treatment with rapamycin (Figure 4a) and quantified (Figure 4b). Our results show that rapamycin treatment of ERRα knockdown, but not control cells, significantly inhibited cell migration. To confirm these findings, MDA-MB-231 control or ERRα knockdown cells were grown in low glucose media and wound healing assay was performed following 14 h treatment with either rapamycin or PF4708671 (Figure 4c) and quantified (Figure 4d). Consistent with the lung colonization assay, MDA-MB-231 sh-ERRα cells exhibited greater migratory capacity than MDA-MB-231 sh-control cells and only sh-ERRα knockdown cell migration was sensitive to rapamycin treatment (Figures 4c and d). Interestingly, the S6K1 inhibitor PF4708671 was able to significantly inhibit migration of both sh-control as well as sh-ERRα cells, indicating that cell migration is uniquely dependent on S6K1.

To further test the ability of rapamycin to affect the transformed phenotype of sh-ERRα cells, a focus formation assay was performed and quantified in MDA-MB-231 control or ERRα knockdown cells grown in reduced serum DMEM (Figures 4e and f). Consistent with our previous data, ERRα knockdown cells were able to form more colonies than sh-control cells, however, unlike control cells, the colony formation ability of ERRα knockdown cells was sensitive to rapamycin treatment. Taken together, our results indicate that although inhibition of ERRα increases the oncogenic potential of ERα-negative cells, it also results in increased S6K1 expression and activity, which renders the cells uniquely sensitive to mTORC1-S6K1 pathway inactivation, leading to overall inhibition of the transformed phenotype.

**Figure 5:**
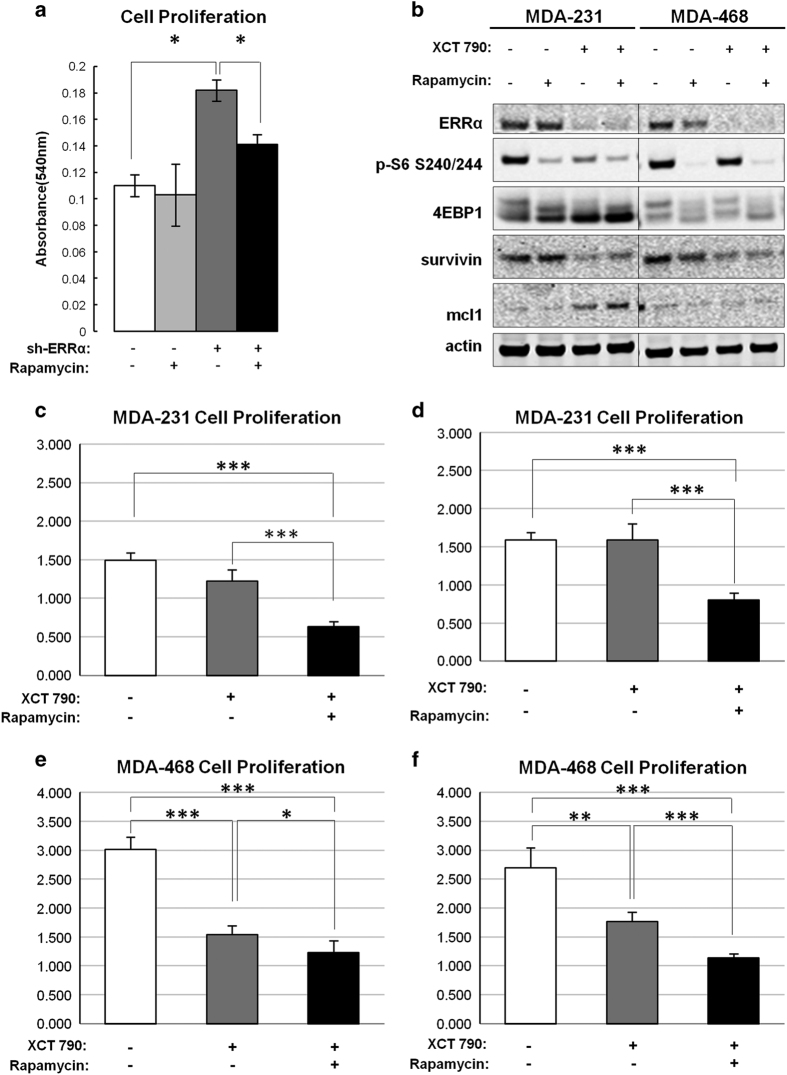
ERRα inhibition sensitizes cells to rapamycin-dependent inhibition of cell proliferation under conditions of S6K1 dependence. (**a**) Stable MDA-MB-231 control cells or ERRα knockdown cells were seeded in 96-well plate in complete DMEM media and allowed to attach overnight. The cells were then treated with 20 nM rapamycin in reduced serum media for 5 days as indicated, and cell proliferation assay was performed as described in ‘Materials and methods’ section. Quantification of the cell proliferation assay was performed and graphed using paired Student’s *t-*test. * represents *P*<0.05. (**b**) MDA-MB-231 and MDA-MB-468 cells were treated with 20 nM rapamycin and 5 μM XCT 790 in reduced serum DMEM, as indicated. Twenty-four hours following treatment, cells were lysed as described in ‘Materials and methods’ section and probed with the indicated antibodies. (**c**) MDA-MB-231 cells were seeded in 96-well plate in complete DMEM media and allowed to attach overnight. The following day, cells were placed in reduced serum media, supplemented with 5 μM XCT 790 alone or in combination with 20 nM rapamycin and cell proliferation assay was performed after 2 days of treatment as described in ‘Materials and methods’ section. Quantification of the cell proliferation assay was performed and analyzed using paired Student’s *t-*test. *** represents *P* <0.001. (**d**) MDA-MB-231 cells were seeded in 96-well plate in complete DMEM media and allowed to attach overnight. The following day, cells were placed in low glucose media, supplemented with 5 μM XCT 790 alone or in combination with 20 nM rapamycin and cell proliferation assay was performed after 2 days of treatment as described in ‘Materials and methods’ section. Quantification of the cell proliferation assay was performed and analyzed using paired Student’s *t-*test. *** represents *P*<0.001. (**e**) MDA-MB-468 cells were treated as described in **c**. * represents *P*<0.05. *** represents *P*<0.001. (**f**) MDA-MB-468 cells were treated as described in **d**. ** represents *P*<0.01. *** represents *P*<0.001.

We next examined the effects of reduced serum culture on cell proliferation as a function of ERRα expression and S6K1 inhibition. Consistent with our data, MDA-MB-231 cells with ERRα knockdown grown in reduced serum media demonstrated statistically significant upregulation of cell proliferation. Importantly, the proliferation of MDA-MB-231 cells with ERRα knockdown cultured under reduced serum conditions become sensitive to rapamycin inhibition (Figure 5a). To validate our findings, MDA-MB-231 and MDA-MB-468 cells were treated with a synthetic reverse agonist of ERRα XCT 790^(ref. 35)^ alone or in combination with rapamycin (Figure 5b). We observed that 24 h treatment with XCT 790 was sufficient to reduce ERRα protein expression and maintain S6K1 pathway inhibition in co-treatment with rapamycin as indicated by reduced S6 protein phosphorylation at S240/244. Interestingly, co-treatment of cells with XCT 790 and rapamycin was synergistic in promoting dephosphorylation of 4EBP1 as evidenced by a more prominent hypophosphorylated (lower) band, further supporting the notion that ERRα inhibition sensitizes ERα-negative cells to mTORC1 inhibitors. Importantly, the combination of XCT 790 and rapamycin was able to promote apoptosis as evidenced by lowered expression of the pro-survival marker survivin and increased expression of the pro-apoptotic marker mcl1.

To further investigate the ability of the combination of XCT 790 and rapamycin to inhibit cell proliferation, MDA-MB-231 and -468 cells were cultured under reduced serum and low glucose conditions, under which the cells depend on the activity of the S6K1 pathway. MDA-MB-231 cells grown under reduced serum conditions and treated with both XCT 790 and rapamycin showed statistically significant downregulation of proliferation compared to either untreated cells or cells treated with XCT 790 alone (Figure 5c). Similar results were observed under low glucose treatment (Figure 5d). A similar effect on cell proliferation was observed with MDA-MB-468 cells (Figures 5e and f), and while these cells were also sensitive to XCT 790 treatment alone, a co-treatment with rapamycin was able to further inhibit cell proliferation. Taken together, our results show that ERRα transcriptionally regulates S6K1 expression rendering ERα-negative cells sensitive to S6K1 pathway inhibitors when used in combination with ERRα inhibitors.

## Discussion

ERα-negative breast cancer accounts for ~10–17% of all breast cancer cases and this cancer subtype tends to be more aggressive and is associated with a poor prognosis in patients. We and others have previously determined that ERRα expression is higher in TNBC and contributes to its pathogenicity.^3,18^ While ERα-positive breast cancers can be treated with endocrine therapy, TNBC is treated with standard chemotherapy due to lack of FDA-approved targeted therapy options. Therefore, there is a need for novel targeted therapies for treatment of TNBC. Moreover, there is a need for reliable biomarkers that would allow us to identify those individuals that would benefit from such treatments.

In the current study, we focused on investigating the mechanisms by which ERRα regulates cell proliferation and survival. Specifically, we found that ERRα negatively regulates S6K1 expression by direct regulation of its gene expression. Knockdown of ERRα triggered increased expression of S6K1 p70 and p85 isoforms, which was mediated by increased *RPS6KB1* gene expression. Increased S6K1 protein expression correlated with sensitivity to either the mTORC1 inhibitor rapamycin or the S6K1 inhibitor PF4708671 under reduced serum or low glucose conditions. This was consistent with a recent report that showed PF4708671-mediated cytotoxic effects in cells undergoing metabolic stress.^36^ It is interesting that relatively modest changes in the levels of the kinase can lead to significantly increased dependence on continued pathway signaling, and lead to dramatic changes in drug sensitivity.

ERα-negative cells express increased levels of ERRα, enabling adaptation and survival even under nutrient-deprived and stress-induced conditions. Our results indicate that inhibition of ERRα expression under reduced serum conditions sensitizes the cells to rapamycin and reduces cell proliferation. Similarly, reduction of ERRα expression sensitized the cells to the S6K1 inhibitor PF4708671 in low glucose conditions. Importantly, the combination treatment of ERα-negative cells using the ERRα inhibitor XCT 790 and rapamycin had a synergistic effect on the reduction of cell proliferation. A recent study showed that pharmacological inhibition of ERRα increased the efficacy of PI3K/mTOR inhibitors.^37^ Our present work provides a plausible biochemical explanation whereby ERRα inhibition results in higher S6K1 expression and mTORC1 inhibitor sensitivity. Finally, cells with reduced ERRα expression were less able to migrate to the lung when treated with rapamycin and we mechanistically link this effect to increased expression of S6K1 in the absence of ERRα, which sensitizes cells to mTOR/S6K1 inhibitors.

Our results reveal an exciting therapeutic opportunity in TNBC patients: combination of ERRα inhibitors with rapamycin, an FDA-approved drug for several oncology indications, including breast cancer, or S6K1 inhibitors, which are in active stages of development.
